# Luft als Element: Literatur, Klima und Thomas Manns *Tod in Venedig*

**DOI:** 10.1007/s41245-021-00133-7

**Published:** 2021-08-30

**Authors:** Eva Horn

**Affiliations:** Institut für Germanistik, Universität Wien, Universitätsring 1, 1010 Wien, Österreich

## Abstract

Das aktuelle Interesse an Phänomenen der Atmosphäre wie Wetter und Klima hat sich bislang vor allem auf die literarische Auseinandersetzung mit der frühen Meteorologie gerichtet. Der vorliegende Aufsatz schlägt eine wissenshistorische und ästhetische Erweiterung der Perspektive vor: ein Blick auf die sehr viel ältere, aber höchst wirkmächtige Tradition, *Luft als Element* zu verstehen. Von der antiken Medizin bis ins späte 19. Jahrhundert ist Luft – als Witterungsphänomen, lokales Klima oder Medium schädlicher Dünste – eine spürbare und intensiv wirksame Dimension von Umwelt, die auf Körper, Seelen und Gesellschaften einwirkt. Diese Tradition wird in der Moderne langsam verdrängt, überdauert aber in der Literatur. Während die Wissenschaften von der Atmosphäre die Luft entsinnlichen, bewahren literarische Texte ein Sensorium für ihre Qualitäten und Wirkmacht, das es neu zu entziffern gilt. Als Beispiel einer solchen meteorologischen Lektüre liest der Aufsatz Thomas Manns Novelle *Der Tod in Venedig* (1912) als einen Text, der sich exakt am Übergang von der alten Theorie der Luft als Element zu einem modernen Verständnis von Atmosphäre und Ansteckung situiert. In den Stimmungen und Witterungen, die der Text entfaltet, zeigt sich Luft als Resonanzraum und maßgeblicher Schrittmacher für Aschenbachs Untergang.

## Abstract (en)

Contemporary interest in atmospheric phenomena like weather and climate has so far focused primarily on the engagement of literature with early meteorology. This essay proposes broadening the perspective both in terms of a history of knowledge and of aesthetics. Its focuses on the much older, yet extremely persistent tradition of understanding the *air as an element*. From Ancient medicine to the late nineteenth century air – as weather, local climate, or medium of noxious vapors – was a tangible and intensely effective dimension of the environment, affecting bodies, souls, and society. While receding in modern science, this tradition survived in literature which insisted on the connection between the air and human senses, affects, and ways of life. While the emerging atmospheric sciences set about desensitizing the air, literary texts preserved a sensorium of its qualities and efficacy which needs to be deciphered anew. This essay provides an example of such a ›meteorological reading‹ with attention to Thomas Mann’s novella *Death in Venice* (1912), situating the text at a moment of transition from the old theory of air as an element to a modern understanding of atmosphere and contagion. Read from this perspective, the moods and weather conditions meticulously unfolded in the text come to the fore and prove to both resonate with and set the pace for Aschenbach’s demise.

## I.

Wind und Wetter, Wolken, Klima, Luft und ganz allgemein atmosphärische Phänomene haben derzeit in der Literaturwissenschaft eine Konjunktur, die sich nicht allein aus der Aktualität des Klimawandels erklären lässt. Dem Interesse an »Phänomenen der Atmosphäre«, wie ein entsprechender Sammelband heißt, geht es dabei einerseits um die Emergenz und Geschichte eines »unsicheren Wissens« über einen komplexen, ephemeren und nicht regelförmigen Gegenstand. Andererseits steht eine Ästhetik im Zentrum des Interesses, die diese flüchtigen Qualitäten und Zustände der Umgebung mit den affektiven, körperlichen, kognitiven oder auch sozialen Zuständen von Subjekten in ein Verhältnis setzt.^1^ Der Atmosphären-Begriff besticht dabei durch seine semantische Dehnbarkeit, der sich vom Naturgegenstand über Witterungsphänomene bis hin zur kollektiven oder individuellen Stimmung erstreckt – und damit eine Vielfalt divergenter Untersuchungsperspektiven bequem abdeckt. Im Kern geht es dabei aber zumeist um metaphorische Übertragungen, in denen eine Semantik des Wetters zur Charakterisierung von Affekten, sozialen Stimmungslagen, geschichtlichen Prozessen, philosophischen Programmen und Ähnlichem dienen kann. »Das Wetter gerät einerseits zum Bildspender für chaotische Zustände aller Art, und andererseits beeinflussen die Krisen gesellschaftlicher Um- bzw. Unordnung den wissenschaftlichen Ausgriff auf das Wetter [...]«, so schreibt etwa Oliver Grill über die Konvergenzen von Meteorologie und Literatur im 19. Jahrhundert.^2^ Auch Claus-Michael Schlesingers Untersuchung über die »Poetik der Meteore im 18. Jahrhundert« zielt ganz auf eine meteorologische Metaphorologie, in der Helligkeit mit Aufklärung, das Wolkige mit dem Unklaren und Verworrenen gleichgesetzt wird.^3^

Ein epistemologisch schärfer konturiertes Programm entwirft die »literarische Meteorologie«, die Michael Gamper am Werk Adalbert Stifters entwickelt hat. Wie Grill geht es ihm vornehmlich um die im 19. Jahrhundert entstehende Wissenschaft der Meteorologie als Form eines »unsicheren Wissens« von den Zukünften des Wetters. Die Schwierigkeit, Wetterprognosen zu stellen, verweist dabei immer auch auf die Schwierigkeit in der Darstellung einer Welt, die einerseits ständigen Ordnungsanstrengungen der Protagonisten ausgesetzt ist, sich diesen andererseits aber immer neu entzieht. Gampers literarischer Meteorologie geht es darum, in der epistemologischen Schwierigkeit des Wetter-Wissens eine poetologische Reflexion des Textes auf die Grenzen des Schilder- und Erzählbaren zu finden: »An der ›literarischen Meteorologie‹ wird [...] zu beobachten sein, wie [Literatur] Aussagen über ihren Gegenstand, das Wetter, macht, es wird stets aber auch zu berücksichtigen sein, wie sie dabei immer auch Bestimmungen über sich selbst, über Dichtung als ästhetisches Darstellungsmedium, trifft.«^4^

So produktiv und innovativ dieses Forschungsprogramm ist, so erscheint mir der wissenspoetologische Fokus auf die Geschichte der Wetter-Prognostik einerseits, auf die metaphorischen Übertragungen und Äquivalenzen zwischen Natur und Kultur, Wetter und menschlichen Dingen (wie Gesellschaft, Geschichte oder Gefühlen) andererseits als eine Verengung des Themas. Sie ist dies sowohl in ästhetischer wie in historischer Hinsicht. Denn das, was wir heute als »Wetter«, »Atmosphäre« und »Klima« fassen, hat eine wissenshistorische und semantische Tradition, die nicht nur viel älter ist als die Anfänge der modernen Meteorologie, sondern die auch literarisch viel breiter wirksam ist. Diese Tradition besteht lange *vor*, aber auch *neben* den modernen Wissenschaften von der Atmosphäre – von der Meteorologie und Klimatologie bis zur Erdsystemwissenschaft, Ökologie und Mikrobiologie. Erst im 20. Jahrhundert wird diese Tradition endgültig als ›veraltet‹ ad acta gelegt, aber sie persistiert noch in Redewendungen, Gesundheitsregeln, Alternativmedizin – und nicht zuletzt in der Literatur. Es ist eine Tradition, die menschliche Körper und Seelen, Gesundheitszustände, soziale Institutionen und Gebräuche mit Naturgegebenheiten wie Landschaften, Temperaturen, Winden und Jahreszeiten in engsten Bezug setzt. Sie figuriert unter vielfältigen Termini: »Klima« (lange ein geografischer und medizinischer Begriff), »Himmelsstrich«, »Luft«, »Witterung«, »Wetter« – und erst spät »Atmosphäre«.^5^ Diese Tradition beruht nicht allein auf metaphorischen Übertragungen oder Ähnlichkeiten zwischen Menschen- und Naturdingen. Vielmehr setzt sie auf Figuren der *Kontiguität*: Durchdringung, Bedingung, Berührung, Affizierung, Stimmung und Formung des Menschen durch die Luft. Wo die modernen Wissenschaften von der Atmosphäre auf Messung, Datensammlung, Beobachtung, Berechnung und Prognose des Wetters setzen, setzt diese ältere Tradition auf eine Vorstellung von *Luft als Element*.

Worum es mir dabei geht, ist eine Erweiterung der Perspektive: Statt nur die Vorgeschichte eines modernen Wissens von der Atmosphäre zu rekonstruieren und damit eine moderne Episteme in die Vergangenheit zurück zu projizieren, erscheint es mir nötig, auch die vormodernen Wissensbestände und Wahrnehmungsformen von Witterungen, klimatischen Bedingungen, Luftzuständen, Winden etc. mit in die Perspektive einzubeziehen. Sie prägen und strukturieren über mehr als zwei Jahrtausende das Verhältnis des Menschen zu seiner allernächsten Umwelt. »Men may live whole days without food, not a moment without air«, bemerkt der Arzt und Satiriker John Arbuthnot 1733 in seinem einflussreichen *Essay on the Effects of Air on Human Bodies*.^6^ Luft, Klima, Witterung ist so die invasivste und existenziellste Dimension menschlicher Umwelt, ein unmittelbares Medium des Lebens.^7^ Diese vormodernen Wissensbestände sind nicht nur höchst heterogen, sondern erscheinen uns auch in einem radikalen Sinn veraltet, unvereinbar mit modernen wissenschaftlichen Paradigmen, aber auch beargwöhnt aufgrund ihrer problematischen politischen Implikationen, etwa der Rede von ›idealen‹ und ›problematischen‹ Klimata im Zuge des Kolonialismus. Sie sprechen davon, dass die Luft (oder das Klima) den Menschen prägt oder verändert, seine körperliche und geistige Gesundheit beeinflusst, seine Stimmungen prägt, politische Institutionen bedingt und kulturelle Unterschiede erklärt. Das heißt auch, dass man, um diesen Diskurs zu verstehen, viele Unterscheidungen aufgeben muss, die zum Kernbestand modernen Denkens gehören: die Differenzierung zwischen Körper und Umwelt, zwischen Angeborenem und Erworbenem, zwischen Natur und Gesellschaft.^8^ In dieser Tradition ist die Luft nicht immaterielles Medium, Wetter und Klima nicht bloßer Hintergrund menschlicher Geschicke und Geschichte, sondern treibende Kraft.

## II.

Eine Geschichte der *Luft als Element* kann hier bestenfalls skizziert werden, schon weil sie aus sehr heterogenen Wissensformationen besteht. Sie reicht von der antiken Elementenlehre zur hippokratischen Medizin und ihren Ausläufern bis ins späte 19. Jahrhundert, durchdringt aber auch die Geografie und damit Kulturtheorien, die »Klima« (in einem vormodernen Sinne von lokalen Umweltbedingungen) als konstitutiv prägend für Kulturen und Gesellschaften betrachten. Diese Tradition externalisiert Luft nicht als etwas dem Menschen und seiner Kultur Äußerliches, sondern bringt die Zustände der Luft und die Art der Landschaft mit der Mentalität, dem Gesundheitszustand und der Lebens- und Staatsform der Bewohner einer Region in direkte Verbindung.^9^ Die Elementenlehre versteht die Welt insgesamt als Mischungsverhältnis der vier Elemente, eine Mischung, die auch den Zustand des menschlichen Körpers und seiner Gesundheit bestimmt. Luft spielt dabei früh eine grundlegende Rolle: Anaximenes bestimmt sie als den Urstoff (*arché*), aus dem die anderen Elemente hervorgehen. Auch das Göttliche entstammt der Luft.^10^ Empedokles formuliert schließlich eine Theorie der vier Elemente (*stoicheía*) als materielle »Wurzeln« (*rhizōmata*) alles Existierenden. In seiner Elementenlehre sind die vier Elemente Luft, Feuer, Wasser und Erde nicht aus einem einzigen Urstoff, sondern gleichursprünglich und gleichrangig. Aus ihnen besteht die Welt, aus ihnen bestehen Körper ebenso wie deren Umgebung.^11^ Ein Außerhalb der Elemente, die durch die Kräfte der Anziehung (*philótēs*) und des Konflikts (*neíkos*) in ständiger Bewegung und Mischung gehalten werden, gibt es nicht. Damit wird eine weitreichende Korrespondenz zwischen dem Menschen (Körper und Seele) und der umgebenden Welt hergestellt: Das Viererschema der Elemente, das den Kosmos ordnet, findet sich im Körper wieder als Harmonie oder Disharmonie der Körpersäfte und Organe. »Der Mensch«, so Hartmut und Gernot Böhme, »ist mitten in der Natur. Darum ist die antike Medizin von Beginn an in weitester Erstreckung konzipiert: denn in Gesundheit und Krankheit verkörpert der Mensch die umgebende Natur; daß er dies tut, ist eben *seine* Natur.«^12^

Die hippokratische Medizin denkt Körper geprägt von »Luft, Wasser und Orten«, so der Titel eines einflussreichen Traktats aus dem *Corpus Hippocraticus*.^13^ Luft- und Wasserqualität, die Art der Böden, die Hauptnahrungsmittel, aber vor allem die klimatischen Bedingungen eines Orts prägen, so Hippokrates, die körperliche Konstitution, die ortstypischen Krankheiten und sogar die Mentalitäten und Gewohnheiten seiner Bewohner. Winde und Witterungen, oder auch der Wechsel der Jahreszeiten bestimmen über Gesundheit und Krankheit, bringen Seuchen und formen die Charaktereigenschaften eines Volkes. So entsteht neben einer medizinischen Theorie des Klimas eine Kulturtheorie der klimatischen Einflüsse. Sie besagt, dass das Klima einer Region – insbesondere die Temperaturen – mit der Mentalität der Einwohner (tapfer oder feige, arbeitsam oder faul, temperamentvoll oder lakonisch etc.) auch ihre Kultur beeinflusst, ihre Religion, ihre Gebräuche, ihre Kunst und sogar, so prominent vorgetragen von Montesquieu, ihre Gesetze und sozialen Institutionen.^14^ »Klima« in einem vormodernen Sinne bindet Kulturen an die Orte und Umwelten, in denen sie sich befinden, und dient zur Erklärung von kulturellen Unterschieden. Das gemeinsame ›Im-Klima-sein‹, die geteilte Luft, stiftet die Identität von Gesellschaften und Kulturen.

Die Wiederbelebung und Weiterentwicklung der hippokratischen Tradition im 17. und 18. Jahrhundert generiert eine besondere Aufmerksamkeit für die gesundheitlichen Wirkungen der Luft. Es entsteht eine ›meteorologische‹ oder ›Umwelt-Medizin‹ avant la lettre.^15^ Ihre Aufgabe ist es, wie schon der Titel von Arbuthnots *Essay on the Effects of Air on Human Bodies* es auf den Punkt bringt, die Wirkungen der Luft auf menschliche Körper (und Seelen) zu studieren. Das bedeutet auch, die schädlichen Einwirkungen der Luft zu verstehen und zu vermeiden. Bestimmte Winde oder Wetterlagen, in der Luft befindliche Dünste und Schwebstoffe, Feuchtigkeit, Temperatur und sogar der Luftdruck werden zur potenziellen Quelle aller denkbaren Krankheiten. Feuchte Luft beispielsweise wird für Koliken, Durchfall und Auszehrung verantwortlich gemacht, kalte für Rheuma, Halsweh und tränende Augen.^16^ Dabei ist die meteorologische Medizin vor allem eine beobachtende und vorsorgende Medizin: Es gilt, sich vor krankmachenden Lüften durch die richtige Kleidung, eine günstige Architektur, regelmäßiges Lüften und viele andere Vorsichtsmaßnahmen zu schützen, noch bevor die Krankheit ausbricht. Ist sie erst da, hilft nur noch Luftwechsel – die Reise an Orte mit gesünderer und reinerer Luft wie Berge und Küsten.

Um die gesundheitlichen Wirkungen der Luft genauer zu verstehen – etwa das Auftreten saisonaler Fieber oder die Korrelation von Krankheitsbildern mit bestimmten Wetterlagen –, beginnen Ärzte und Privatpersonen, Wetteraufzeichnungen anzulegen, in ihre Tagebücher zu integrieren oder in Briefen ausführlich über das Wetter zu berichten. Von Goethe, Lichtenberg, Georg Forster, Novalis und Stifter bis hin zu Raabe, Hofmannsthal und Nietzsche finden wir ausführliche Notizen zur Witterung als Protokolle der jeweiligen Tagesverfassung.^17^ Wetterbeobachtung wird Selbstbeobachtung, der Körper, wie Jan Golinski formuliert, zum »menschlichen Barometer«.^18^ Im Zentrum steht dabei die individuelle Empfindlichkeit gegenüber den Einwirkungen der Luft, nicht nur im Hinblick auf die körperliche Verfassung, sondern auch auf die Zustände der Seele und des Geistes. Meteorologische Medizin ist eine Medizin der Konstitution: Sie blickt auf den individuellen Körper als robust oder empfindlich, wetterfühlig oder in bestimmten Klimata zu spezifischen Krankheitsbildern neigend. Es geht immer um die Konstellation von individueller Körperlichkeit und Witterung, eine Konstellation, die noch für das Verständnis von Tuberkulose und ihrer Heilung durch »Luftkuren« maßgeblich sein wird.

Das wohl folgenreichste Konzept in dieser Tradition ist der Begriff »Miasma«. Miasmen sind schädliche Ausdünstungen des Bodens, stehender Gewässer oder lokaler Fäulnisprozesse. Wörtlich bedeutet »Miasma« »Befleckung« – und kann in diesem Sinne sowohl eine materielle Verschmutzung als auch eine moralische Schuld meinen.^19^ In beiden Fällen bringt Miasma Krankheiten – insbesondere Epidemien. Der berühmteste Fall ist die Pest, die Theben mit der ungesühnten Schuld ihres Königs Ödipus befällt. Miasma ist ebenso eine menschliche Verschuldung wie eine Verpestung der Luft. Wichtig ist, dass diese »Befleckung« kollektiv wirkt, eine Erkrankung der Gesellschaft, nicht des Einzelnen. Miasmen machen Menschen krank, die die gleiche Luft atmen – und erklären so den Ausbruch lokaler Epidemien, wie schon ein Hippokrates argumentiert: »But when an epidemic of one disease is prevalent, it is plain that the cause is not regimen but what we breathe, and that this is charged with some unhealthy exhalation.«^20^

Fast alles kann Miasmen ausstoßen. Sie sind Ausdünstungen des Todes wie des Lebens. Sie entstehen durch Verwesung und Fäulnis, steigen aus Wasserflächen, Sümpfen und Abwässern auf, werden durch stinkende Industrien wie Gerbereien, Abdeckereien oder Brauereien erzeugt, sind die Ausdünstungen von Kranken oder die Atemluft des Nachbarn. Auf dem Land sind es die fetten Böden, Flussufer oder die Exkremente und Exhalationen der Tiere, die Miasmen hervorbringen. In der Stadt rühren die schädlichen Dünste vom engen Zusammenleben der Menschen, wie eine Bemerkung Adalbert Stifters illustriert: »[...] unsere Väter bauten hie und da so enge Gassen, daß es in manchen geschieht, daß, wenn ich morgens mein Fenster öffne, um frische Luft hereinzulassen, ich mir die Nachtluft aus der Schlafkammer meines Nachbars gegenüber hereinfange, der ebenfalls geöffnet hat und mir Morgen wünscht.«^21^ In der Miasmen-Theorie ist die Luft Medium von Krankheiten, Gerüchen, aber auch einfach von zu großer sozialer Nähe.

Innerhalb dieser Vorstellung von Krankheitsgenese muss es also darum gehen, die Entstehung schädlicher Exhalationen zu unterbinden, etwa durch Ausräuchern verpesteter Räume, durch Säubern und Lüften, durch das Trockenlegen von Sümpfen und die Auslagerung geruchsintensiver Orte aus den Städten. Schon im 18. Jahrhundert beginnt man daher, die Quellen schädlicher Ausdünstungen zu entfernen oder durch Reinigungsprozesse unschädlich zu machen. Friedhöfe, Gerbereien und Abdeckereien werden aus den Innenstädten verbannt, Krankenhäuser und Kanalisation aufwändig gereinigt. Die Hygiene-Bewegung des 19. Jahrhunderts wird diese Bemühungen um ›reine‹ Luft dann auf Städtebau und Architektur, aber auch auf individuelle Gesundheitsvorsorge ausdehnen. Eine besondere Ausprägung dieser Sorge um zuträgliche Luft in der zweiten Hälfte des 19. Jahrhunderts ist schließlich die Entwicklung von »Luftkurorten« mit besonders frischer und reiner Luft, seien es Berge oder Meeresküsten, in denen sich der Organismus von den Anfechtungen ungesunder Lüfte erholen kann.

So ist *Luft als Element *sehr viel mehr als Wetter, Klima oder Atmosphäre in einem modernen Sinn. Sie verbindet die Menschen mit dem Ort, an dem sie sich aufhalten, sie ist die Qualität eines Orts. Als genius loci ist Luft Quelle von Krankheit und Gesundheit, aber beeinflusst auch von Sitten und Lebensweisen. Dabei ist sie immer auch ein Medium des Sozialen: Sie verbindet Menschen mit anderen Menschen und Tieren, aber auch das Lebendige mit dem Kranken und Toten. Bemerkenswert ist, dass die Zustände der Luft auf intensive Weise *sinnlich wahrnehmbar* sind: Luft ist spürbar, sichtbar, fühlbar und natürlich intensiv riechbar. Alain Corbin hat anschaulich beschrieben, wie die neue Aufmerksamkeit gegenüber den schädlichen Wirkungen der Luft im 18. Jahrhundert zu einer ganz neuen Sensibilität – fast Überempfindlichkeit – gegenüber Gerüchen führt. Man kann, so die Vorstellung, an Gestank sogar sterben. Aber auch spüren kann man die Luft: Die »Fasern« des Körpers, so argumentieren schon Arbuthnot und Montesquieu, reagieren unmittelbar auf Wärme und Kälte der Umgebung. Bei Kälte ziehen sie sich zusammen und geben dem Körper mehr Spannkraft, bei Wärme entspannen sie sich, und der Körper erschlafft.^22^ Auch Feuchtigkeit wirkt erweichend auf das Körpergewebe und schwächt daher.^23^ Nicht zuletzt sind die Zustände der Luft auch sichtbar. Luft zeigt sich in den Wolken und Dünsten, in Witterungen und Lichtstimmungen, aber auch zunehmend in Form von Ruß, Rauch und Dampf, die die Landschafts- und Himmelsmalerei des 19. Jahrhunderts prägen.

Die Vorstellung von Luft als Element ist so verbunden mit einer *Aisthesis* der Witterungen, Atmosphären, Klimata, Luftqualitäten. Sie basiert auf einer sinnlichen Wahrnehmbarkeit der Luft, die trainiert und wissenschaftlich aufgezeichnet werden kann, aber auch von individueller Sensitivität abhängt. Luft als Element wird mit allen Sinnen wahrgenommen: gerochen, empfunden, gesehen, erlitten, verinnerlicht oder abgewehrt. Sie wirkt nicht nur individuell, sondern auch kollektiv auf ganze Gesellschaften und Kulturen. Luft als Element ist so im höchsten Maße sinnlich und affektiv wirksam. Menschliche Körper und Seelen sind die Resonanzkörper ihrer Schwingungen.

## III.

Heute empfinden wir Luft als »immateriell«, unspürbar, selbst in ihren pathogenen Formen. Luftverschmutzung, Feinstaub, Klimawandel und Krankheitserreger kann man nicht sehen, riechen, spüren. Für ein modernes Verständnis von Luft sind ihre Störungen – von der globalen Erwärmung bis zur Übertragung des Virus SARS-CoV-2 – sinnlich nicht wahrnehmbar. Luft ist immateriell, was wir von ihr wissen, wissen wir durch Messungen und Modellierungen. »The air is unique among the elements in […] signifying the being of non-being, the matter of the immaterial,« schreibt Steven Connor.^24^ Dabei wird vergessen, dass dies noch im 19. Jahrhundert völlig anders ist. Luft war alles andere als »immateriell«, sie war im Gegenteil intensiv spürbar und ebenso gefährlich wie wohltuend. Wie, so muss man sich fragen, konnte die Luft in dieser Weise verschwinden, leer und »immateriell« werden? Wie konnte sie zum stummen, bestenfalls pittoresken Hintergrund menschlicher Existenz werden? Luft ist heute zum abstrakten Gegenstand wissenschaftlicher Vermessung und Berechnung geworden, sei es in der Meteorologie, in der Klimatologie oder Atmosphärenchemie oder auch in einer Umwelt-Medizin, die sich an nicht wahrnehmbaren Schadstoffen abarbeitet.

Was ist passiert? Wie wurden Luft, Witterung, Klima so immaterialisiert, dass wir nur noch durch die Vermittlung der Wissenschaft Zugang dazu haben? In der Moderne, ziemlich genau um 1900, verschwindet allmählich die Vorstellung von Luft als Element. Die Ideen über Miasmen, schädliche Winde oder ungesunde Klimata weichen einem Begriff von ›Atmosphäre‹, die sich gerade durch ihre Unspürbarkeit auszeichnet. Was in der Luft schwebt und wirkt – wie einst die Miasmen oder Witterungen –, wird zu einem von der Luft selbst unabhängigen, externalisierten Agens. Das hat mit zwei Prozessen zu tun: Einerseits dem Aufkommen einer datengestützten, hochgradig abstrakten Meteorologie und Klimawissenschaft, die in der zweiten Hälfte des 19. Jahrhunderts beginnt, Luft auf ihre Chemie und Physik zu reduzieren, sie zugleich aber auch zum ersten Mal als globales Strömungssystem zu vermessen. Mit einem extremen Aufwand an Datenerhebung und -standardisierung wird die Atmosphäre nun als regelhaftes, wenngleich hochkomplexes System beschreibbar.^25^ War »Klima« im alten Sinne ein lokales Phänomen, wird die »Atmosphäre« nun zu einem Gegenstand, der nur noch global zu erfassen ist und mit Körpern und Kulturen nichts mehr zu tun hat.

Alexander von Humboldt ist eine Figur, die sich genau an diesem Übergang zwischen einem alten und einem modernen Verständnis von Klima situieren lässt. Einerseits definiert er »Klima« in seinem Spätwerk *Kosmos* noch ganz im alten Sinne: »Klima bezeichnet in seinem allgemeinsten Sinne alle Veränderungen in der Atmosphäre, die unsere Organe merklich afficiren...«.^26^ Andererseits schafft er mit seiner Isothermenkarte die Grundlage für eine Erfassung des Klimas als globalem Systemzusammenhang, eine Perspektive, die Klima als Ortsprinzip und körperlich spürbare Größe schließlich auflösen wird. Von nun an wird das Wissen von der Luft zur Meteorologie – der Erfassung und Auswertung von Wetterdaten zum Zwecke der Wettervorhersage. Ganz gegen seine jahrhundertealte Bedeutung als geografischer und medizinischer Begriff wird Klima nun gänzlich abstrakt gefasst – als Durchschnitt der Witterungen:

Klima ist die Summe der meteorologischen Erscheinungen, die den mittleren Zustand der Atmosphäre an irgendeiner Stelle der Erdoberfläche kennzeichnen. [...] Das Klima ist die Gesamtheit der ›Witterungen‹ eines längeren oder kürzeren Zeitabschnittes, wie sie *durchschnittlich* zu dieser Zeit des Jahres einzutreten pflegen.^27^

Dies ist die berühmte Definition des österreichischen Meteorologen Julius von Hann, des Begründers der modernen Klimatologie. Als Durchschnittsgröße entzieht sich Klima von nun an der sinnlichen Wahrnehmung und verschwindet als »average weather« in den Hochleistungsrechnern moderner Klimaforschung.^28^

Andererseits verdankt sich die Immaterialisierung der Luft als medizinische Größe der Ablösung der Miasmen-Theorie durch die Mikrobiologie. Statt schädlicher Dünste werden nun identifizier- und isolierbare Mikroorganismen für die Ansteckung und Ausbreitung von Infektionskrankheiten verantwortlich gemacht. Die Theorie der Ansteckung durch Keime, die am Ende des 19. Jahrhunderts beginnt, die Miasmen-Theorie zu verdrängen, trennt Krankheitserreger vom Medium Luft. Sie verweist auf ganz andere Übertragungsmedien wie Wasser, Nahrungsmittel, Körperflüssigkeiten, kontaminierte Gegenstände oder Mücken – und damit auch auf andere Formen der Infektion, vom Essen und Trinkwasser bis hin zur menschlichen Berührung. Luft ist damit nur noch eines von vielfältigen Übertragungsmedien; worauf es ankommt, ist nurmehr ein Erreger, der nicht anders als unter dem Mikroskop dingfest zu machen ist. Lange Zeit ist Luft nicht einmal mehr einer der wichtigsten Übertragungswege, bis sie neuerdings im Zeichen des durch Aerosole übertragenen Virus SARS-CoV‑2 zu neuer Prominenz gelangt. Das bewirkt auch eine Neuorganisation des Wissens über die Luft. Als Theorie ist die Miasmenlehre buchstäblich ›wolkig‹. Sie geht stets vom Zusammentreffen vielfältiger Faktoren aus: den Zuständen der Luft, den in ihr schwebenden Dünsten, aber auch der Konstitution und Lebensgewohnheiten des Patienten. Diese komplexe und diffuse Theorie weicht mit der Keimtheorie einer präzisen und monokausalen Ätiologie durch einen spezifischen Erreger. Damit verschwindet die Luft aus den Sinnen. Denn die Affizierung durch ein krankmachendes Agens ist nicht mehr spürbar wie im Gestank, im heißen Wind oder in feuchter und trüber Luft. Im Gegenteil steckt das Dämonische der Keime gerade darin, dass sie nicht wahrzunehmen sind, sondern nur noch durch die Maschinerie medizinischer Apparaturen nachzuweisen – vom Mikroskop bis zur Laborkultur. So wird die Luft »leer«, eine neutrale Trägersubstanz, chemisch analysierbar, ablösbar von all den Ingredienzien oder Qualitäten, die sie mit sich bringt. Unspürbar, unsichtbar, gewichtslos, ein lebloses Gemisch von Gasen.

Was hat diese Geschichte der *Luft als Element* nun mit Literatur zu tun? Während die Wissenschaft sich daran macht, die Luft zu entmaterialisieren und in Messdaten aufzulösen, ist es die Literatur, so meine These, in der die Reichhaltigkeit und Sinnlichkeit dieser Tradition noch lange weitergedacht und ästhetisch inszeniert wird. Die Literatur bewahrt, wenn man so will, die *Aisthesis der Luft*, ihre sinnliche Wahrnehmbarkeit, ihre Wirkung auf Körper und Psyche, ihre Rolle als Faktor, der Kulturen und Gesellschaften prägt – kurz: das, was ihr die meteorologischen Wissenschaften auszutreiben suchen. Zugleich setzt sie sich aber auch intensiv mit diesem epistemischen Wandel auseinander. In dieser Auseinandersetzung zeigt sich ein ästhetischer Widerstand gegen die Bemühung, die Aisthesis der Luft in Daten und Wetterprognosen, ihre Spürbarkeit und pathogene Wirksamkeit in der Rekonstruktion von Infektionswegen aufzulösen. So verfolgt etwa Goethe zwar sehr genau die frühen Bemühungen zur Erzeugung von meteorologischen Messdaten, schreibt Gedichte auf Luke Howards Klassifikation der Wolken und entwirft einen eigenen *Versuch zur Witterungslehre*.^29^ Dennoch ist diese Auseinandersetzung, wie die Forschung gezeigt hat, vor allem eine subtile *Kritik* an der wissenschaftlichen Klassifikation und Typisierung eines amorphen und ephemeren Gegenstands wie Wolken oder Witterung. Der Wolken-Klassifikation stellt Goethe eine Morphologie im Übergang, in ständiger Wandlung begriffener Gestalten entgegen.^30^ Die datengestützte Erfassung von Witterungsphänomenen korrigiert er durch eine Emphase der sinnlichen Anschauung und »höheren Aufmerksamkeit«, die sich nicht auf Klassifikation und Begrifflichkeit beschränkt.^31^

Ein anderer Zeitgenosse der beginnenden Wetterforschung ist Adalbert Stifter. Auch bei ihm zeigt sich eine Faszination für die wissenschaftliche Erfassung, verbunden mit einem Widerstand, der das Vermessungswesen der Meteorologen entweder lächerlich macht oder ihr eine anthropologische und ästhetische Dimension von Wetter entgegensetzt.^32^ Den Lektüren einer »literarischen Meteorologie« bei Stifter, der es um die wissenspoetologische Auseinandersetzung mit den entstehenden Wetterwissenschaften geht, muss so eine literarische Klimatologie – im alten Sinne von ›Klima‹ – an die Seite gestellt werden. Hier geht es nicht mehr um Wetterprognostik, sondern um das, was Klima mit dem Menschen und der Mensch mit dem Klima macht. In etlichen seiner Texte behandelt Stifter diese Verbindung von Landschaften und Lebensformen oder erzählt, etwa in *Brigitta* oder *Zwei Schwestern*, von der moralischen Wirkung gefährlicher oder unwirtlicher Lüfte auf seine Protagonisten.^33^

Die Persistenz einer Tradition der Luft als Element zeigt sich ferner in der Fülle von Wettereinträgen und -beobachtungen in zahlreichen Tagebüchern und Briefen von Schriftstellern. Extreme körperliche und seelische Empfindlichkeit gegenüber der Witterung ist noch um 1900, wie Patrick Ramponi zeigt, ein »Epochenleiden«.^34^ Nietzsches Briefe beispielsweise sind voller Klagen über seine gesundheitlichen Beschwerden durch Witterung und Klima, die er mit Überlegungen zur Diätetik und Hygiene verbindet.^35^ Wilhelm Raabe beginnt jeden seiner Tagebucheinträge mit einem kurzen Wetterbericht. Fontanes Wanderungen durch die Mark Brandenburg sind ebenso wie sein Romanwerk voller Beobachtungen über Gerüche, gesunde oder verpestete Lüfte, und über die Luft als Element seiner berühmtesten Protagonistin, Effi Briest.^36^ Nicht zuletzt zelebrieren Reise- und Tropenromane – von Robert Müllers *Tropen* (1915) bis zu Christoph Ransmayrs *Die Schrecken des Eises und der Finsternis* (1984) über die Payer-Weyprecht-Expedition in die Arktis 1872-74 – ausführlich die enorme Wirkung, die extremes Klima auf den Körper, die Wahrnehmung, vor allem aber die seelische und sexuelle Verfassung nicht ›akklimatisierter‹ Europäer hat. Umgekehrt werden der Luftkurort und das Sanatorium zu einem paradigmatischen literarischen Schauplatz, der die Frage von Krankheit und Gesundung zu einer Frage des richtigen Klimas oder der individuellen Akklimatisierung macht.^37^

## IV.

Thomas Mann ist ein Autor, der diese Tradition der Luft als Element – sowohl in ihren medizinischen wie klimaanthropologischen Dimensionen – in besonderer Weise aufgreift, um sie gut informiert gegen die zeitgenössische Wissenschaft in Stellung zu bringen. Schon seine frühe Erzählung *Tristan* (1902) ist in einem Luftkurort angesiedelt, dessen Kälte und stille Luft allerdings der affektiven Überhitzung seiner Protagonisten nichts entgegenzusetzen hat. Und sein großer Sanatoriums-Roman *Der Zauberberg* lässt sich – gegen den Strich der üblichen Lektüren als Bildungs- oder Zeitroman, Gesellschaftsstudie der endenden Belle Époque oder philosophische und politische Reflexion – natürlich auch klimatheoretisch verstehen: als Geschichte eines besonders extremen Falls von Akklimatisation eines Flachländers im nur vermeintlich gesunden, de facto physisch und psychisch pathogenen Klima des Berghofs. Aber das wäre eine andere Untersuchung.

Stattdessen möchte ich einen kurzen, aber prominenten Text Manns als Modell lesen, an dem sich die Verwebung der Luft als Element mit seinem ästhetischen Verfahren zeigen lässt: die Novelle *Der Tod in Venedig* (1912).^38^ Der Text siedelt sich exakt an der Schwelle zwischen dem alten, ›elementaren‹ Verständnis von Luft und den neuen Paradigmen der Bakteriologie an, die die Luft immaterialisieren werden. Oft genug wurde er als Künstlernovelle, ästhetische Selbstreflexion Thomas Manns, Behandlung verbotener homoerotischer Neigungen oder auch als Auseinandersetzung mit Nietzsches *Geburt der Tragödie* verstanden.^39^ Ich möchte ihn dagegen einer Lektüre unterziehen, die das in den Vordergrund rückt, was gemeinhin als Hintergrund und Ausschmückung überlesen wird: die Witterungen. Die in fünf Kapiteln wie eine Tragödie aufgebaute Novelle erzählt die Geschichte Gustav von Aschenbachs, eines gealterten Schriftstellers, der sich bei einer Reise nach Venedig in einen vierzehnjährigen Knaben verliebt, ihm nachstellt, sich (vermutlich) mit der Cholera infiziert und stirbt. So gesehen, wäre die Erzählung eine über Ansteckungen – sowohl mit einer epidemischen Krankheit als auch mit einem verbotenen, rauschhaften Begehren.^40^ Betrachtet man die Geschichte jedoch im Licht einer meteorologischen Lektüre, so erscheint sie plötzlich als Protokoll der Wirkmacht von Witterungen, Jahreszeiten und schädlichen Ausdünstungen auf einen besonders wetterfühligen Protagonisten. Denn Aschenbach, dessen Stimmungen, Ideen und zunehmende erotische Ekstase die Erzählung minutiös wiedergibt, ist ein klassischer Patient der meteorologischen Medizin.

Das bedeutet, sich weniger an den Reisestationen Aschenbachs, seinen gelehrten Assoziationen, Phantasien und Träumen oder auch den überdeutlichen ›Botenfiguren‹ zu orientieren, die ihn auf Schritt und Tritt als Präfiguration alles Kommenden begleiten. Es bedeutet vielmehr, wie Hans Ulrich Gumbrecht in einer kurzen, aber wegweisenden Skizze vorgeschlagen hat, sich an die wechselnden Witterungen zu halten und an die Stimmungen, die diese erzeugen.^41^ Der meteorologische Plot der vielinterpretierten Novelle hat denn auch nichts mit Knabenliebe, Nietzsche oder ästhetischer Theorie zu tun. Er geht so: Der wetterfühlige Aschenbach reist von einer unnatürlichen und ungesunden Jahreszeit in München über Umwege an einen ebenfalls ungesunden, von schädlichen Winden und üblen Ausdünstungen heimgesuchten Ort, das frühsommerliche Venedig. In Venedig durchlebt er verschiedene Witterungen: schwüle Wärme, strahlenden Sonnenschein und schließlich drückenden Scirocco mit fauliger Luft. Diese Wetter begleiten seinen exaltierten Zustand und treiben ihn voran. Nach einem kraftlosen Fluchtversuch, zerrüttet von zu viel Sonne, schädlichen Dünsten und heißen Winden, erliegt er schließlich seiner wetterempfindlichen Konstitution am Strand, schutzlos der Witterung und offenen Unendlichkeit des Meeres ausgeliefert.

Wie etliche Erzählungen über die Gefahren ungewohnter, vornehmlich warmer Klimata ist auch dieser Witterungs-Plot eng mit einer Reisegeschichte verknüpft. Es beginnt schon in München mit einem für die Jahreszeit unüblichen Wetter: »Es war Anfang Mai und, nach naßkalten Wochen, ein falscher Hochsommer eingefallen. Der Englische Garten [...] war dumpfig wie im August [...]«. (501) Schon diese jahreszeitliche Irregularität des Münchner Klimas verweist auf Krankheit. So schildert schon Hippokrates in seiner Abhandlung über Epidemien ausführlich die jeweiligen jahreszeitlichen Vorgeschichten solcher Krankheitsausbrüche.^42^ In diesem ungewöhnlichen Wetter begegnet Aschenbach – markiert durch die Todessymbolik des Friedhofs – eine erste Botenfigur, nicht zufällig im »Wetterkragen«: ein ihn scharf anblickender Fremder mit »gekreuzten Füßen« wie der Putto, der in der Antike die Doppelfigur von Schlaf/Tod darstellt (503).^43^ Der Fremde weckt in Aschenbach zwar keine Todessehnsucht, aber etwas, das ihn zuletzt in den Tod führen wird, nämlich anfallsartige »Reiselust«. Begleitet wird diese von der Vision einer exotischen, überdeutlich erotisierten Landschaft in tropischem Klima, ein Genrebild aus der Kolonialliteratur:

er sah, sah eine Landschaft, ein tropisches Sumpfgebiet unter dickdunstigem Himmel, feucht, üppig und ungeheuer, eine Art Urweltwildnis aus Inseln, Morästen und Schlamm führenden Wasserarmen, – sah aus geilem Farrengewucher, aus Gründen von fettem, gequollenen und abenteuerlich blühendem Pflanzenwerk haarige Palmenschäfte nah und ferne emporstreben ... und fühlte sein Herz pochen vor Entsetzen und rätselhaftem Verlangen. (504)

Diese Vision – so weiß man am Schluss der Novelle – nimmt die wichtigsten Motive der Geschichte vorweg: Eine hochgradig sexualisierte Landschaft (als solche wird sich Venedig für Aschenbach dann erweisen), Hitze und quellendes, aber auch sich zersetzendes Leben (die Kanäle und die schlechte Luft Venedigs), und nicht zuletzt die Gefahr, die von dieser zugleich verlockenden und gefährlichen Landschaft ausgeht. Natürlich zeigt diese schon ganz am Anfang des Geschehens evozierte Tropenlandschaft auch jene »warmen Moräste des Ganges-Deltas« (578), aus dem historisch die Cholera 1911 nach Venedig wanderte.^44^ Noch bevor Aschenbach überhaupt aufgebrochen ist, verweisen ein Todesbote und eine exotisch-gefährliche Landschaft im tropischen Klima auf seinen Tod am Strand, von dem eine »respektvoll erschütterte Welt« (592) Wochen später hören wird. So ist die ganze Novelle eingeklammert in klimatische Vorzeichen, die zugleich Vorzeichen des Todes sind.

Aschenbach mit seiner von Anfang an als kränklich und angespannt geschilderten Konstitution lebt nicht nur ein Leben des disziplinierten, auf klassische Formen und gehobene Sprache bedachten Schriftstellers. Er lebt auch ein strenges Regime der Klima-Disziplin. Den größten Teil des Jahres verbringt er in München, die Sommer aber in einem Haus in den Bergen, wo die warme Jahreszeit eher kühl-regnerisch ausfällt. Das von September bis Mai gemäßigte Klima Münchens wird im Sommer, wenn es warm wird, mit einem frischen Bergklima vertauscht. Bergluft gilt schon der meteorologischen Medizin als besonders gesund und rein, und sie schenkt Aschenbach genau die nordisch-heroische Kühle, der er sich auch in seiner Kunst verschrieben hat.

Es ist daher ein radikaler Bruch mit der Aschenbach’schen Klima-Disziplin, im späten Mai ausgerechnet nach Italien – also den Süden – zu fahren. Genau das transportiert auch die Tropen-Vision, die seine »Reiselust« einleitet. Sie spielt nicht nur auf den Ursprungsort der Cholera in Indien an, sondern sie ist Bergklima mit umgekehrten Vorzeichen. Tropen-Klima ist – davon zeugen ganze Bibliotheken kolonialmedizinischer Traktate – schlechthin ungesundes, Körper und Seele zerrüttendes Klima.^45^ Die Reiselust, von Anfang an beängstigend und lustvoll zugleich, ist also die Lust nach einem *gefährlichen Klima*, nach genau jener Atmosphäre der Sinnlichkeit und Krankheit, die das tropische Klima ausmacht. Dabei spielen nicht nur Dünste, Miasmen und der »mephitische Odem« (578), aus dem die Cholera aufsteigt, eine Rolle, sondern auch die geografische Lage selbst. Warmes Klima, so behauptet die jahrhundertealte Klimatheorie, erzeugt Erschlaffung, Überempfindlichkeit, Schwäche, aber auch Sinnlichkeit und moralische Lockerung. Denn Wärme macht hedonistisch, schlaff und empfänglich für Sinnesreize, sie lenkt alles Sinnen und Trachten ins Erotische. So schreibt schon Montesquieu:

In den kalten Ländern hat man wenig sinnliche Empfänglichkeit für Vergnügungen; sie ist größer in den gemäßigten Ländern, außerordentlich in den warmen Ländern. Wie man die Klimate nach den Breitengraden unterscheidet, könnte man sie sozusagen nach den Graden der sinnlichen Empfänglichkeit unterscheiden. [...] Bei dieser Feinheit der Organe, wie man sie in den heißen Ländern besitzt, wird das Gemüt vorzüglich durch alles erregt, was auf die Vereinigung der beiden Geschlechter Bezug hat, alles ist darauf bezogen.^46^

Aschenbach ist ein Bewohner des klimatischen Nordens, kontrolliert, sittsam, pflichtbewusst. Er sucht die Kühle und Kälte als klimatisches Pendant seines Wesens. Nicht zufällig sind die Protagonisten seines schriftstellerischen Werks, allen voran der Preußen-König Friedrich II., geprägt von einer Tapferkeit, die »in stolzer Scham die Zähne aufeinanderbeißt und ruhig dasteht, während ihr die Schwerter und Speere durch den Leib gehen.« (511) So ist denn auch Tapferkeit und kontrolliertes Ertragen von Schmerz eine der hervorstechendsten Eigenschaften der Völker des Nordens, so Montesquieu, dessen XIV. Kapitel seiner Rechtstheorie *Vom Geist der Gesetze* (1748) zur wichtigsten Grundlage einer neuzeitlichen Vorstellung von der Verbindung von Klima und Mentalität geworden ist.^47^ Sehenden Auges begibt sich Aschenbach also in ein Klima, das ihm nicht nur fremd, sondern – wie er sogar weiß – gänzlich unzuträglich ist. Dies zeigt sich schon bei der meteorologisch enttäuschenden Ankunft in Venedig bei trübem Wetter, das auch so bald nicht besser wird.

Jedes der drei Kapitel, die in Venedig spielen, ist nun geprägt von einer anderen Wetterlage und damit auch einer anderen Atmosphäre, in die Aschenbach eintritt und die ihn in zunehmendem Maße affiziert. Was als klimatische Deplatzierung beginnt, wird nicht nur, wie es immer wieder heißt, zur Tragödie einer ›Entwürdigung‹, sondern auch zur zunehmenden körperlichen und seelischen Zerrüttung des Protagonisten. Diese aber wird grundiert und vorangetrieben durch Atmosphären oder, mit Gumbrecht, »Stimmungen«, in denen »die physische Umwelt in der leichtesten, am wenigsten aufdringlichen Weise einen Körper berührt.«^48^ Schon die symbolisch überdeutliche Motivkonstellation des Anfangs verweist auf etwas, das die Erzählung gleichsam im Hintergrund oder in der Latenz des Plots trägt: Konstellationen und Resonanzverhältnisse von Witterungen, Gerüchen, Lichtverhältnissen und Affekten. Diese Stimmungen entstehen durch Blicke, Gesellschaft, Gesten, Aussehen, Musik, Düfte und Geräusche und treiben die wechselnden Zustände des Protagonisten voran. Als solche Konstellationen entsprechen diese Atmosphären sehr genau dem zugleich komplexen und diffusen Verständnis von Luft in der meteorologischen Medizin. So betrachtet, erscheint die Novelle als eine »Erzählung der Stimmungen«, also »Befindlichkeiten des Gemüts, die nicht in der Verfügung des durch sie affizierten Individuums liegen.«^49^ Witterungen und ihre Wirkung auf Emotion und Körpergefühl des Menschen, aber auch Musik wären damit Paradigmen einer solchen Affizierung: ein Gefühl, das einen gleichsam ›von außen‹ ergreift. Präziser fasst David Wellbery – mit Blick auf die reichhaltige semantische Tradition des Begriffs der Stimmung – den Kern des Konzepts in drei verschiedenen Dimensionen: Stimmungen sind (erstens) emotionale Zustände und weisen somit »Ichqualität« auf, allerdings ohne auf das Innenleben beschränkt zu sein. Vielmehr sind sie (zweitens) auch »Atmosphären, die uns umgeben«, Umwelten. Sie bestehen im Zusammenspiel mehrerer Elemente, ohne an einzelne Objekte gebunden zu sein, sondern sind ein Zusammenklingen von Emotion und Umgebung. Und sie teilen sich (drittens) mit, sind ansteckend und umfassen so mehrere Subjekte, die sich in einer gemeinsamen – von ihnen zugleich erzeugten und erlittenen – Stimmung befinden.^50^ Diese Stimmungen zu lesen, bedeutet, nicht so sehr den Reise- und Ansteckungswegen zu folgen, sondern die Momente einer Korrespondenz von innerem Gefühl und äußerer Umgebung zu entziffern. Diese Korrespondenzen von Gefühl und Umwelt folgen nicht einfach den Stadien von Aschenbachs zunehmender Leidenschaft und liefern gleichsam deren Hintergrund. Vielmehr *organisieren* sie sein Selbstverhältnis, sein Körpergefühl, seine Launen, seinen Gesundheitszustand, seine Wahrnehmungen und sogar seine sozialen Kontakte, die ihrerseits hochgradig atmosphärisch geschildert sind: etwa in der Darstellung des zweifelhaften Dampfschiffs, das ihn nach Venedig bringt, in der Atmosphäre des Hotels am Lido und besonders in jener von alarmierenden Gerüchen, Gesten, Blicken, Musik und Gefühlen getragenen Sänger-Szene im fünften Teil. Am sinnfälligsten jedoch wird diese Korrespondenz in der Witterung, die dabei durchaus nicht einfach die Gefühlslage des Protagonisten spiegelt, sondern sie vielmehr hervorbringt, antreibt, hemmt oder beflügelt.

## V.

Betrachten wir also die Stimmungen in der Novelle am Leitfaden der Witterungen. Bei der Ankunft zeigt sich Venedig zunächst bewölkt, warm, und still, ein unbestimmtes, gleichsam abwartendes Wetter. Aber schon in der unheimlichen Gondelfahrt zum Hotel wird Aschenbach »lau angerührt vom Hauch des Scirocco«. (524) Im Hotel empfangen ihn »stark duftende Blumen« und ein Blick aufs offene Meer (528), aber schon bald scheint das Wetter Aschenbach wieder vertreiben zu wollen: Es herrscht »Landwind«, eben Scirocco, »unter fahl bedecktem Himmel lag das Meer in stumpfer Ruhe« und Aschenbach spürt den »fauligen Geruch der Lagune«, von dem ihn sogleich »Verstimmung« befällt. (533) Bereits hier wird dem empfindlichen Aschenbach selbst klar, dass diese Luft für ihn nicht zuträglich ist. Er erinnert sich an einen Aufenthalt, bei dem »sein Befinden so schwer geschädigt [wurde], daß er Venedig wie ein Fliehender hatte verlassen müssen« (ebd.). Zudem peinigt ihn das, was schon Stifter an der Stadtluft beklagt: die Dünste des sozialen Lebens, die »Gerüche, die aus Wohnungen, Läden, Garküchen quollen, Öldunst, Wolken von Parfum und viele andere Schwaden [...] Zigarettenrauch [...]« (541).

Dabei ist der immer wieder erwähnte Scirocco ein Wind, dessen Wirkung vor allem darin liegt, dass er die Seele quält. Fast zeitgleich mit Manns Novelle entsteht Christian Morgensterns Sonett »Scirocco« (1911)*, *das die Gefährdung durch die eigenen Gefühle, der Aschenbach schließlich erliegen wird, präzise auf den Punkt bringt:

**Scirocco**
Ja, wer so immer wüßte, was ihn quält,
wie heute, da der laue Südwind brütet
und jeden Geist, der sich nicht streng behütet,
wie einen roten Docht erstickt und schwält.
An solchen Tagen wird, wie man erzählt,
in Süditalien, wenn das Messer wütet,
dem Rasenden ein Teil der Pön vergütet
und seine Schuld der Schuld des Sturms vermählt.
Wohl dem, der stärker als solch Fieber ist,
womit oft Erde sich und ihn verstört,
wohl dem, der’s klar erkennt und niederzwingt.
Und wehe, wer nur einen Tag vergißt,
wie sehr er dunklen Mächten gleich gehört,
wenn er nicht unablässig wacht und ringt.^51^

Der Wind erscheint hier wie in der meteorologischen Medizin als die physische, atmosphärische Verkörperung einer unbestimmten, dafür umso gefährlicheren Anfechtung des Körpers wie der Seele, die Erschütterung des Menschen durch ein diffuses Naturphänomen, gegen das es sich zu schützen und zu wappnen gilt. Die beiden Quartette beschreiben die Problemlage. Der Scirocco ist ein schwer fassbares Übel, atmosphärisch wie ein »brütender« Druck, etwas Quälendes, das von außen kommt, aber im Inneren seine Wirksamkeit entfaltet. Morgenstern benutzt das Bild des Brennens und Schwelens: Der Wind erstickt den Geist wie einen brennenden Docht zum flammenlosen, aber umso hitzigeren Nachglimmen. Die äußeren Folgen solchen Schwelens, so das zweite Quartett, sind Gewalt, Zornesausbrüche, Messerstechereien – nicht zufällig in »Süditalien«, einer Region, die wie Manns Venedig alle Züge des klimatischen Exotismus trägt. Und natürlich hat man dort, wie schon Montesquieu weiß, die passenden sozialen Einrichtungen: Scirocco ist der Wind der Unzurechnungsfähigkeit, menschliche Schuld immer auch die Schuld des Sturms. So zeichnet Morgenstern den Wind als eine »Verstörung«, ein »Fieber«, das Mensch und Natur gleichermaßen erfasst. Die Lehre aus dieser Problemlage und ihr Antidot präsentieren die beiden abschließenden Terzette des Sonetts: Es gilt, die Irritation zu erkennen und »niederzuzwingen«. Die Macht der Witterung muss anerkannt werden. So ist Heilung das Ergebnis einer bewussten Entscheidung, einer Haltung. Gegen die Wirkungen des Windes kann man sich nur wappnen, indem man ihre ständige Präsenz anerkennt, ihren Eingebungen nicht nachgibt.

Aber Aschenbach, der sein Leben lang unablässig wachte und rang, ergibt sich nun willig den Wirkungen des Wetters: »Trägheit fesselte den Geist, indes die Sinne die ungeheure und betäubende Unterhaltung der Meeresstille genossen.« (539) Sein folgender, von ihm selbst halb sabotierter Fluchtversuch wird bereits durch die Faszination für Tadzio vereitelt, während er sich noch weismacht, es sei die »Atmosphäre der Stadt, diese[r] leis faulige Geruch von Meer und Sumpf« (545), den er plötzlich mit Genuss einatmet. Denn Tadzio selbst ist die Emanation dieser Witterungs-Stimmung, dieser betörenden venezianischen Luft: » [...] schön wie ein zarter Gott, herkommend aus den Tiefen von Himmel und Meer« entsteigt er dem »Element« (539). Und so nimmt die Tragödie ihren Lauf.

Das vierte Kapitel, in dem sich Aschenbach immer tiefer in seine rauschhafte, aber mit klassischen Zitaten ins Idealische gehobene Verliebtheit hineinsteigert, beginnt mit einer geradezu jubilatorischen Wetterschilderung:

Nun lenkte Tag für Tag der Gott mit den hitzigen Wangen nackend sein gluthauchendes Viergespann durch die Räume des Himmels, und sein gelbes Gelock flatterte im zugleich ausstürmenden Ostwind. Weißlich seidiger Glanz lag auf den Weiten des träge wallenden Pontos. Der Sand glühte. (549)

Gemessen am kühlen, gewitterreichen Wetter in seinem üblichen Sommerwohnsitz empfindet Aschenbach das Klima in Venedig nun als »elysisches Land«, sich selbst »an die Grenzen der Erde« entrückt, »wo leichtestes Leben den Menschen beschert ist, wo nicht Schnee ist und Winter, noch Sturm und strömender Regen, sondern immer sanft kühlenden Anhauch Okeanos aufsteigen läßt und in seliger Muße die Tage verrinnen, mühelos, kampflos und ganz nur der Sonne und ihren Festen geweiht.« (550) Der Text ruft hier nicht nur die alten Wetter-Allegorien auf, sondern auch die Topoi der antiken Klimatheorien: die Vorstellung eines elysischen, ausgewogenen, ›mittleren‹ Klimas, nicht zu kalt und nicht zu heiß, das dem Menschen ideale Lebensbedingungen und der dort ansässigen Kultur eine geistige Blüte verschafft.^52^ In dieser »enthusiasmierenden«, göttlich beseelten Witterung gelingt es Aschenbach, in ständiger Kontemplation Tadzios am Strand zu arbeiten und sich zu Reflexionen über Schönheit, Form und Platons *Phaidros* aufzuschwingen (554). Im jubilatorischen Glanz des idealen Wetters erscheint ihm seine Leidenschaft für den Jungen unter idealischen Vorzeichen, er verklärt Tadzio zur Muse und zum sokratischen Gesprächspartner. Er versucht, »beim Schreiben den Wuchs des Knaben zum Muster zu nehmen, seinen Stil den Linien dieses Körpers folgen zu lassen, der ihm göttlich schien [...]« (556). Aber schon in diesem sonnendurchfluteten Kapitel tendiert auch Aschenbachs Höhenflug nicht nur zur Leichtigkeit sondern jener Leichtfertigkeit, die Bewohnern südlicher Gefilde in der Klimatheorie nachgesagt wird.

So schreibt der Arzt, Psychologe und Wettertheoretiker Willy Hellpach, ein Zeitgenosse Thomas Manns, über das südliche Klima:

Je im Nordteil eines Erdraums überwiegen die Wesenszüge der Nüchternheit, Herbheit, Kühle, Gelassenheit, der Anstrengungswilligkeit, Geduld, Zähigkeit, Strenge, des konsequenten Verstandes-und Willenseinsatzes – je im Südteil die Wesenszüge der Lebhaftigkeit, Erregbarkeit, Triebhaftigkeit, der Gefühls- und Phantasiesphäre, des behäbigeren Gehenlassens oder augenblicklichen Aufflammens. Innerhalb einer Nation sind ihre nördlichen Bevölkerungen praktischer, verläßlicher, aber unzugänglicher, ihre südlicheren musischer, zugänglicher (gemütlicher, liebenswürdiger, gesprächiger), aber unbeständiger.^53^

Und so weicht Aschenbachs bewunderte »Zucht« der »Zügellosigkeit« (557), was sich in den Einwirkungen des Wetters auf seinen Körper zeigt. Er bräunt, wird gestärkt vom «Salzhauch« des Meeres, und »ließ nun alles, was Sonne, Muße und Meerluft ihm an täglicher Kräftigung zuführten, hochherzig-unwirtschaftlich aufgehen in Rausch und Empfindung.« (558) Die Dämmerung, der Wind, die Wolken, das Aufgehen der Sonne über dem Meer werden anthropomorph stilisiert: Götter und Göttinnen erscheinen, begleitet von »kindlichen Wolken« und »Amoretten im rosigen, bläulichen Duft« (559). Die Witterung wird zur Erscheinungsform dessen, was im Inneren Aschenbachs vorgeht: eine Stimmung, in der nicht mehr die Umwelt den Körper und die Psyche berührt, sondern eine Projektionsbewegung, in der wie beim jungen Goethe die innere Gestimmtheit sich auf die Phänomene der Umwelt legt. Was meteorologische Erscheinung ist, wird im erhitzten Geist Aschenbachs zum Aufzug der Götter und der polnische Junge ihm zu Hyakinthos, dem Geliebten Apollons. Und so endet die sich zwischen Wetter und Psyche aufschaukelnde Stimmung schließlich im überwältigten Flüstern »Ich liebe dich!« (563).

Das alles wird begleitet von Gerüchen. Wo das ekstatische vierte Kapitel im »nächtlichen Duft der Pflanzen« endet, beginnt der fünfte Teil – in der Tragödie die Katastrophe – mit ernüchternd-warnendem Gestank: »einem süßlich-offizinellen Geruch, der an Elend und Wunden und verdächtige Reinlichkeit erinnerte.« (563) Mit diesem Geruch beginnen auch die Lügen über den Zustand der Stadt und die sich ausbreitende, aber offiziell geheim gehaltene Cholera-Epidemie. Der weiträumige Einsatz des Desinfektionsmittels Karbol wird darum von den Stadtbewohnern auf die Lüfte geschoben: »Diese Witterung drückt, der Scirocco ist der Gesundheit nicht zuträglich.« (ebd.)

Mit dem Cholera-Ausbruch wendet sich Aschenbachs Leidenschaft zunehmend ins Düstere: Er verfolgt Tadzio und seine Familie durch die Stadt, träumt von orgiastischen Dionysos-Feiern, tauscht verstohlene Blicke mit dem Angebeteten, lässt sich kosmetisch behandeln und beginnt schließlich zu fiebern. Die Ansteckung mit dem »Übel«, wie die Epidemie von allen Beteiligten schamhaft genannt wird, konvergiert mit Aschenbachs »Entnervung und völliger Demoralisation«.^54^ Aber wie sein Aufschwung wird auch Aschenbachs Niedergang von atmosphärischen Phänomenen begleitet und vorangetrieben. Und so lässt sich fragen, ob es wirklich die Cholera ist, an der Aschenbach am Ende verstirbt. Ein Held alten Typs, wäre es ebenso plausibel, dass er an den Anfechtungen des Wetters stirbt, dem er sich in seiner ganzen Leidenschaft willenlos hingegeben hat.

Bemerkenswert ist nämlich, wie Manns Erzählung an dieser Stelle die zwei unterschiedlichen Erklärungen für den Zustand der Stadt gegeneinander führt: den Scirocco einerseits – die Cholera andererseits. Die Ausführungen des Clerks im englischen Reisebüro, der Aschenbach endlich über die »wahren« Verhältnisse aufklärt, erscheinen dabei als Klartext, die Rede vom Scirocco als verlogene Ausflüchte. Die Stadt leidet an einer Cholera-Epidemie. Wie ein versierter Epidemiologe doziert der Angestellte über die Verbreitungsgeschichte der Cholera und ihre Ursprünge in jenem fernen Gangesdelta, berichtet von der Ausbreitung der Krankheit in der Stadt und ihren Übertragungswegen über Nahrungsmittel und Wasser und schildert mit betonter Drastik das Krankheitsbild.

In diesem Diskurs wird das zeitgenössische epidemiologische, bakteriologische und medizinische Wissen über die Cholera akkurat ausgebreitet. Beim Abfassen der Novelle hatte sich Thomas Mann, wie Thomas Rütten gezeigt hat, ausführlich über die zuerst von Robert Koch identifizierten »Vibrionen« informiert, die Erreger der Cholera.^55^ Vorgetragen wird so ein präzises Bild der Keimtheorie von Ansteckung auf dem Wissensstand von 1911. Dreißig Jahre zuvor hatte Robert Koch mit der Anzüchtung des Cholera-Bakteriums die von den englischen Ärzten John Snow und William Budd vertretene These bewiesen, dass die Krankheit von einem lebenden Mikroorganismus im Trinkwasser, *vibrio comma*, ausgelöst wird. Die Popularität des großen Bakteriologen und Hygienikers konnte Thomas Mann selbst im Jahr 1911 bei seiner Reise nach Venedig auf der Adria-Insel Brioni (heute Veliki Brijun) beobachten, wo ein Denkmal an Kochs erfolgreichen Kampf gegen die Malaria auf der Insel erinnert.^56^ Mann, der mit seiner Frau Venedig just beim Cholera-Ausbruch vom Frühling 1911 besuchte, wird vermutlich dort selbst Zeuge der Gerüchte und Vertuschungsversuche der Behörden.

Und so lässt er seinen englischen Aufklärer ganz im Sinne der Keimtheorie die Herkunft und Übertragung der Krankheit erklären – womit das ganze Gerede vom Scirocco und der drückenden Luft als reine Schutzbehauptung entlarvt wird. Nicht diffuse Dünste oder Winde, sondern isolierbare Organismen übertragen die Cholera durch kontaminiertes Trinkwasser oder Lebensmittel. All dies ›weiß‹ die Novelle. Es ist die Rede davon, dass »Nahrungsmittel infiziert worden [waren], Gemüse, Fleisch oder Milch« (579), die Stadtväter warnen »vor dem Genusse von Austern und Muscheln, auch vor dem Wasser der Kanäle« (564). Und natürlich legt die Erzählung verschiedene Infektions-Spuren, wenn sie Aschenbach sowohl einen Granatapfelsaft mit Soda (571) als auch überreife Erdbeeren (587) verzehren lässt.

So ist man also spätestens im fünften Teil nicht mehr in der Theorie der Luft als Element, sondern durchaus auf dem Stand des zeitgenössischen Infektionswissens, dessen Klartext die Miasmen- und Wind-Theorie als reine Vertuschung entlarvt. Die diffuse, umweltbezogene und elementare Genese von Krankheiten wird nicht nur als veraltet abgestempelt, sondern auch als verantwortungslose Schönfärberei einer geldgierigen Tourismusindustrie. Vom neuesten Stand der Wissenschaft aus betrachtet, ist die Rede von Miasmen und Winden zwar sinnfällig, aber nichts als ein Irrtum, hinter dem sich die Wirksamkeit unsichtbarer Erreger verbirgt. Die Lüfte werden zur Lüge.

Aber vielleicht sollte es zu denken geben, dass dieser Sendbote des modernen Infektionswissens für die herrschende Witterung denkbar ungeeignet gekleidet ist: mitten im drückendsten Venezianischen Sommer trägt er Wollsachen (577). Die Infektionstheorie ist eben doch nur die *eine* Seite der Wirklichkeit, die witterungs-vergessene. Denn am Ende, während Aschenbach bereits fiebert, ballt sich auch das Wetter zu einer bedrohlichen Szenerie zusammen, die die innere Zerrüttung Aschenbachs gleichsam in der Atmosphäre veräußerlicht.

Lauwarmer Sturmwind war aufgekommen; es regnete selten und spärlich, aber die Luft war feucht, dick und von Fäulnisdüften erfüllt. Flattern, Klatschen und Sausen umgab das Gehör und dem unter der Schminke Fiebernden schienen Windgeister üblen Geschlechts im Raume ihr Wesen zu treiben [...] (586)

Das Wetter treibt sein Wesen: am Ende bleiben die Lüfte, Witterungen, Gerüche und Stimmungen die heimlichen Impulsgeber für Aschenbachs Schicksal. Nicht umsonst umfängt den Sterbenden am Strand eine Stimmung von »Herbstlichkeit, Überlebtheit« (590). Schutzlos den Witterungen preisgegeben, am Rande der Unendlichkeit, weist sein »lieblicher Psychagog« (592) Tadzio den einsamen, verlorenen Aschenbach hinaus in einen offenen Raum der Elemente, in dem Himmel und Wasser, Luft und Erde sich vereinen.

Thomas Manns *Tod in Venedig* markiert eine tiefgreifende kultur- und wissenshistorische Schwelle: den Übergang von einer Theorie der Luft als Element hin zu einem wissenschaftlichen Paradigma der Infektion, das die Luft als leeres, immaterielles Medium hinter sich lässt. Aber Mann bleibt mit Raffinesse bei einer Ambivalenz zwischen beiden Paradigmen, auch wenn er sie zeitweise als Klartext vs. Lüge gegeneinander führt. Seine Erzählung gewinnt ihre Dynamik und Suggestivität eben nicht aus einer Rekonstruktion von Infektionswegen, sondern aus der Inszenierung von Stimmungen. In diesen Stimmungen, in der filigranen Ausgestaltung von Witterungen und Atmosphären, konvergieren die Entwürdigung Aschenbachs und die Fragwürdigkeit der Stadt Venedig. Eine literarische Subjektivität entfaltet sich hier in eine atmosphärische Umwelt hinein. Eine vibrierende, sich ständig wandelnde, sinnlich überwältigende Umgebung findet ihren Widerhall in den Wandlungen und Ekstasen einer erotischen Obsession. So erzählt Manns Novelle nicht nur vom Niedergang Aschenbachs, sie erzählt auch vom Niedergang einer jahrtausendealten Vorstellung von Luft als Element. Aber sie erinnert noch einmal, gerade im Duktus des Erzählens, an deren überwältigend sinnliche, affektive und ästhetische Macht. Nur scheinbar – und im Modus milder Ironie über das Durchblickertum des Clerks – wird die alte Aisthesis der Luft von neueren wissenschaftlichen Erkenntnissen als Irrtum beiseite gefegt. In der Art seiner Darstellung, aber auch in der Natur seines Protagonisten feiert der Text vielmehr gerade die Macht der Luft, indem sie Aschenbach, den wetterfühligen, aber kontrollierten Klassiker, als ihren Spielball und Opfer zeigt. In der Literatur überdauert so die alte Theorie der Luft als Element. Denn Literatur kann mit der Aisthesis der Luftzustände eine Ästhetik der Stimmungen entfalten, die die Wissenschaften ihr längst ausgetrieben haben. Mit dieser Austreibung wird Luft das, was sie für uns heute ist: ein leeres, immaterielles Medium, ein Gasgemisch und Strömungsgefüge, dessen Komplexität nur noch mit schwerem wissenschaftlichem Gerät beizukommen ist.

## Footnotes

1 Urs Büttner, Ines Theilen (Hrsg.), *Phänomene der Atmosphäre. Ein Kompendium literarischer Meteorologie*, Stuttgart 2017.

2 Oliver Grill, *Die Wetterseiten der Literatur. Poetologische Konstellationen und meteorologische Kontexte im 19. Jahrhundert*, München 2019, 11.

3 Claus-Michael Schlesinger, *Aufklärung und Bewölkung. Poetik der Meteore im 18. Jahrhundert*, Göttingen 2018.

4 Michael Gamper, »Literarische Meteorologie. Am Beispiel von Stifters *Das Haidedorf*«, in: Georg Braungart, Urs Büttner (Hrsg.), *Wind und Wetter. Kultur – Wissen – Ästhetik*, Paderborn 2017, 265–281, hier: 267.

5 Zur Begriffsgeschichte von »Atmosphäre« siehe Craig Martin, »The Invention of Atmosphere«, *Studies in History and Philosophy of Science* 52 (2015), 44–54. Zu einer Theorie von »Luft« als gemeinsamer Nenner dieser heute getrennten, historisch aber oft synonym gebrauchten Begriffe siehe Eva Horn, »Air as Medium«, *Grey Room* 73 (Fall 2018), 6–25.

6 John Arbuthnot, *An Essay Concerning the Effects of Air on Human Bodie*s, London, 1733, 2.

7 Vgl. dazu Horn (Anm. 5) und Eva Horn, »Winds, Miasma, Pollution. Pathologies of the Air as an Environing Medium«, Manuskript im Review.

8 Fabien Locher, Jean-Baptiste Fressoz, »Modernity’s Frail Climate: A Climate History of Environmental Reflexivity«, *Critical Inquiry* 38/3 (Spring 2012), 579–598, hier: 581.

9 Einen Überblick über diese frühen Konzeptionalisierungen von »Klima« bieten Lucian Boia, *The Weather in the Imagination*, übs. Roger Leverdier, London 2005, und Clarence J. Glacken, *Traces on the Rhodian Shore. Nature and Culture in Western Thought from Ancient Times to the End of the Eighteenth Century*, Berkeley, Los Angeles 1966.

10 Anaximenes, »Fragmente und Zeugnisse«, in: Laura Gemelli Marciano (Hrsg.), *Die Vorsokratiker*, Berlin, Boston 2011, I, 72–85.

11 Empedokles, »Fragmente und Zeugnisse«, in: Laura Gemelli Marciano (Hrsg.), *Die Vorsokratiker*, Berlin, Boston 2013, II, 138–317, insb. 166–169.

12 Gernot Böhme, Hartmut Böhme, *Feuer, Wasser, Erde, Luft. Eine Kulturgeschichte der Elemente*, 3. Aufl., München 2014, 166.

13 Hippokrates, *Über die Umwelt *[eigentlich: Über Luft, Wasser, Orte], hrsg. und übers. von Hans Diller, Berlin 1999 (= Corpus medicorum Graecorum 1,1,2).

14 Prominent wurde diese schon aus der Antike stammende Klimatheorie durch das XIV. Kapitel von Montesquieus *Vom Geist der Gesetze*.

15 Die Forschung spricht zumeist von »environmental medicine«, was diese begrifflich aber der modernen »Umweltmedizin« annähert, mit der sie wenig zu tun hat. Mir geht es hier um eine spezifische historische Formation, die die *Gefährdungen des Körpers durch die Luft* ins Zentrum stellt. Daher spreche ich von »meteorologischer Medizin«, die aber auch klimatische Bedingungen, Luftverschmutzung oder individuelle Wetterfühligkeit umfasst. Vgl. Ludmilla Jordanova, »Earth science and environmental medicine: The synthesis of the late Enlightenment«, in: Ludmilla Jordanova, Roy Porter (Hrsg.), *Images of the Earth. Essays in the history of the environmental sciences*, 2. Aufl., Oxford 1997, 127–152. Ebenso bei Vladimir Jankovic, *Confronting the Climate: British Airs and the Making of Environmental Medicine*, New York 2010.

16 Jankovic (Anm. 15), hier: 16.

17 Vgl. Patrick Ramponi, »Wetterfühligkeit und Diätetik. Skizzen zur literarischen Wissensgeschichte eines kulturellen Symptomleidens 1800/1900«, in: Karin Becker, Vincent Moriniaux, Martine Tabeaud (Hrsg.), *L’alimentation et le temps qu’il fait*, Paris 2015, 39–56.

18 Jan Golinski, *British Weather and the Climate of Enlightenment*, Chicago, London 2007, 143.

19 Jacques Jouanna, »Air, Miasma and Contagion in the Time of Hippocrates and the Survival of Miasmas in Post-Hippocratic Medicine«, in: Ders., *Greek Medicine from Hippocrates to Galen. Selected Papers*, hrsg. Philip van der Eijk, Leiden 2012, 121–136, hier: 122.

20 Hippocrates, »Nature of Man«, in: Hippocrates, Heracleitus, *Nature of Man. Regimen in Health. Humours. Aphorisms. Regimen 1–3. Dreams. Heracleitus: On the Universe*, übers. W. H. S. Jones, Bd. IV, Loeb Classical Library 150, Cambridge/MA 1931, 27.

21 Adalbert Stifter, »Wiener Wetter«, in: Ders., *Wien und die Wiener*, Wien 2005, 151.

22 Arbuthnot (Anm. 6), hier: 56. Dieser Text liefert die physiologische Grundlage von Montesquieus Überlegungen zu Klima und sozialen Institutionen.

23 Arbuthnot (Anm. 6), hier: 61.

24 Steven Connor, *The Matter of Air: Science and Art of the Ethereal*, London 2010, 30.

25 Paul Edwards, *A Vast Machine. Computer Models, Climate Data, and the Politics of Global Warming*, Cambridge/MA 2010.

26 Alexander von Humboldt, *Kosmos. Entwurf einer physischen Weltbeschreibung* (1845), hrsg. Otmar Ette, Oliver Lubrich, Frankfurt a. M. 2004, I, 166.

27 Julius von Hann, *Handbuch der Klimatologie* (1883), 3. Aufl., Stuttgart 1908, III, 1.

28 So die Definition der WMO, http://www.wmo.int/pages/prog/wcp/ccl/faq/faq_doc_en.html.

29 Luke Howard, »On the modifications of clouds«, *The Philosophical Magazine* XVI (1803), 97–107. Johann Wolfgang von Goethe, »Howard Trilogie«, in: Ders., *Die Schriften zur Naturwissenschaft*, Weimar 1970, I/11, 233–240.

30 Gerhard Neumann, »Wolkenspuren. Goethes Erfindung einer Poetik des Übergänglichen«, in: Roland Borgards, Almuth Hammer, Christiane Holm (Hrsg.), *Kalender kleiner Innovationen: 50 Anfänge einer Moderne zwischen 1755 und 1856. Für Günther Oesterle*, Würzburg 2006, 303–317. Christian Begemann, »Wolken. Sprache. Goethe, Howard, die Wissenschaft und die Poesie«, in: David Wellbery, Gerhard Neumann (Hrsg.), *Die Gabe des Gedichts. Goethes Lyrik im Wechsel der Töne*, Freiburg i.Br. 2008, 225–242.

31 Johann Wolfgang von Goethe, »Dispositionen der Atmosphäre«, in: Ders., *Schriften zur Naturwissenschaft*, Weimar 1970, I/11, 201. Vgl. dazu die Studie von Claus-Michael Schlesinger (Anm. 3), Kap. VII.

32 So etwa in Stifter (Anm. 21), 145–165.

33 Vgl. Eva Horn, »Stifters Klimatologie. Die Kultivierung der Luft und des Menschen«, *Colloquia Germanica* 53/4 (2021), 323–348.

34 Ramponi (Anm. 17), 50.

35 Vgl. Gregory Moore, »Nietzsche, Medicine, and Meteorology«, in: Gregory Moore, Thomas H. Brobjer (Hrsg.), *Nietzsche and Science*, London, New York 2016, 71–90.

36 Hans J. Rindisbacher, »Effi Briest: Daughter of the Air«, in: Ders., *The Smell of Books: A Cultural-Historical Study of Olfactory Perception in Literature*, Ann Arbor 1992, 86–97. Evi Zemanek, »(Bad) Air and (Faulty) Inspiration. Elemental and Environmental Influences on Fontane«, in: Caroline Schaumann, Heather Sullivan (Hrsg.), *German Ecocriticism in the Anthropocene*, New York 2017, 129–145.

37 Vgl. Vera Pohland, *Das Sanatorium als literarischer Ort: medizinische Institution und Krankheit als Medien der Gesellschaftskritik und Existenzanalyse*, Frankfurt a. M. 1984.

38 Im Folgenden zitiert nach Thomas Mann, *Frühe Erzählungen 1893–1912* (Große kommentierte Frankfurter Ausgabe), hrsg. Terence J. Reed, Frankfurt a. M. 2004, II/1, Seitenzahlen im Text. Ich danke Julia Enzinger für Hinweise und Gespräche über Thomas Mann. Eine eingehendere Lektüre der Novelle wird Teil ihres Dissertationsprojekts zu Klimakonzeptionen in Literatur und Wissenschaft zwischen 1890 und 1940 sein.

39 Neuere Ansätze, die gleichwohl den klimatisch-meteorologischen Bedeutungsstrang weitgehend ignorieren, finden sich in Stefan Börnchen, Georg Mein, Gary Schmidt (Hrsg.), *Thomas Mann. Neue kulturwissenschaftliche Lektüren*, München, Paderborn 2012. Interessante Überlegungen zum medizinhistorischen Subtext der Novelle finden sich in: Sabine Meine u.a. (Hrsg.), *Auf schwankendem Grund. Dekadenz und Tod im Venedig der Moderne*, München, Paderborn 2014.

40 So die Lektüre von Elisabeth Strowick in: *Sprechende Körper – Poetik der Ansteckung. Performativa in Literatur und Rhetorik*, München 2009, 281–288.

41 Hans Ulrich Gumbrecht, »Scirocco. Über Stimmung und Modernität von Thomas Manns ›Der Tod in Venedig‹«, in: Stefan Börnchen, Claudia Liebrand (Hrsg.), *Apokrypher Avantgardismus. Thomas Mann und die klassische Moderne*, München 2008, 299–306, hier: 300. Gumbrechts kurze, aber scharfsichtige Skizze zu den Witterungen sieht die fünf Kapitel der Novelle mit jeweils einer dem Zustand Aschenbachs korrespondierenden Witterung ausgestattet. De facto ist es aber doch komplizierter: verschiedene Witterungen und unterschiedliche Diskursformen über Wetter und Klima weben ein Netz wiederkehrender Motive durch den gesamten Text. Dieses Netz von Motiven verklammert den Anfang im schwülen München über die Tropen-Vision eng mit der aus den Tropen stammenden Cholera in Venedig im 5. Teil.

42 Hippocrates, »Epidemics I«, in: Ders., *Ancient Medicine. Airs, Waters, Places. Epidemics 1 and 3. The Oath. Precepts. Nutriment. *Bd. 1, übers. W. H. S. Jones, Loeb Classical Library 147, Cambridge, MA 1923, 147.

43 Zu den Todesbildern in der Novelle vgl. Günther Blamberger, »Kippfiguren. Thomas Manns Todesbilder«, in: Meine (Anm. 39), 37–48.

44 Vgl. zu den kolonialen Subtexten dieser Tropen-Schilderung Amrita Ghosh, »The Horror of Contact. Understanding Cholera in Mann’s *Death in Venice*«, *Transtext(e)s Transcultures*, 12 (2017), 1–10.

45 Der Klassiker zu den Gefahren tropischer Klimata ist James Johnson, *The Influence of Tropical Climates on European Constitutions*, 2. Aufl., London 1818.

46 Charles-Louis de Secondat, Baron de Montesquieu, *Vom Geist der Gesetze* (1748), hrsg. und übers. von Ernst Forsthoff, Tübingen 1992, 313.

47 Montesquieu (Anm. 46), 311, 313.

48 Gumbrecht (Anm. 41), 301.

49 Gumbrecht (Anm. 41), 300 f.

50 David Wellbery, »Stimmung«, in: Karlheinz Barck u.a. (Hrsg.), *Ästhetische Grundbegriffe*, Stuttgart, Weimar 2003, V, 704 f.

51 Christian Morgenstern, *Ich und Du. Sonette, Ritornelle, Lieder *(1911), hrsg. Heinrich Proskauer, Basel 1973, 17.

52 Aristoteles, *Politik*, VII.7.1327a24–36.

53 Willy Hellpach, »Kultur und Klima«, in: Heinz Wolterek (Hrsg.), *Klima – Wetter – Mensch*, Leipzig 1938, 429 f. Das wichtigste Werk Hellpachs, eine Summe des medizinisch-meteorologischen Wissens der Zeit, erscheint im gleichen Jahr wie Manns *Tod in Venedig*: Willy Hellpach, *Die geopsychischen Erscheinungen. Die Menschenseele unter dem Einfluß von Wetter und Klima, Boden und Landschaft*, Leipzig 1911.

54 So in Thomas Manns Arbeitsnotizen, in Terence J. Reed (Hrsg.), *Der Tod in Venedig. Text, Materialien, Kommentar*, München 1983, 120.

55 So zeigen seine Arbeitsnotizen ausführliche Exzerpte zum 1911 aktuellen Wissensstand, Reed (Anm. 54), 107–114. Vgl. auch Thomas Rütten, »Cholera in Thomas Mann’s Death in Venice«, *Gesnerus* 66/2 (2009), 256–287.

56 Die biografischen Grundlagen der Novelle und die Reise Thomas Manns und seiner Frau nach Venedig 1911 rekonstruiert sehr exakt Rütten (Anm. 55).

